# Enzymatic ligation of an antibody and arginine 9 peptide for efficient and cell-specific siRNA delivery

**DOI:** 10.1038/s41598-021-01331-1

**Published:** 2021-11-08

**Authors:** Yu Ando, Hikaru Nakazawa, Daisuke Miura, Maho Otake, Mitsuo Umetsu

**Affiliations:** Department of Biomolecular Engineering, Graduate School of Engineering, Tohoku University, Aoba 6-6-1, Aramaki, Aoba-ku, Sendai, 980-8579 Japan

**Keywords:** Molecular engineering, Nanobiotechnology, Biotechnology, Molecular biology

## Abstract

A fusion protein comprising an antibody and a cationic peptide, such as arginine-9 (R9), is a candidate molecule for efficient and cell-specific delivery of siRNA into cells in order to reduce the side effects of nucleic acid drugs. However, their expression in bacterial hosts, required for their development, often fails, impeding research progress. In this study, we separately prepared anti-EGFR nanobodies with the K-tag sequence MRHKGS at the C-terminus and R9 with the Q-tag sequence LLQG at the N-terminus, and enzymatically ligated them in vitro by microbial transglutaminase to generate Nanobody-R9, which is not expressed as a fused protein in *E. coli*. Nanobody-R9 was synthesized at a maximum binding efficiency of 85.1%, without changing the binding affinity of the nanobody for the antigen. Nanobody-R9 successfully delivered siRNA into the cells, and the cellular influx of siRNA increased with increase in the ratio of Nanobody-R9 to siRNA. We further demonstrated that the Nanobody-R9–siRNA complex, at a 30:1 ratio, induced an approximately 58.6% reduction in the amount of target protein due to RNAi in mRNA compared to lipofectamine.

## Introduction

RNA interference (RNAi) is a cellular mechanism for post-transcriptional gene regulation mediated by small interfering RNA (siRNA)^1, 2^. Specific gene silencing by siRNA holds significant promise for providing new treatment strategies in a wide range of diseases, including cancer and viral infections^3^. Thus, siRNA-based technology is attractive owing to its target gene specificity, relatively low siRNA immunogenicity, and simple design^4^. However, it has four limitations: (1) As siRNA has no cell-type specificity, its medical application would require high doses resulting in high cost; (2) it may cause side effects^4, 5^; (3) because of their hydrophilicity, negative charge, and large molecular weight, siRNA molecules cannot readily cross the cell membrane^6, 7^; and (4) siRNA are subject to rapid renal clearance and degradation by endogenous RNases and can be recognized by the innate immune system^8–10^.

To overcome these limitations, recent studies have focused on the combination of a target-specific antibody and a cationic peptide with siRNA^11–16^. An antibody is an immune system-related biomolecule that can bind a specific region on an antigen^17^. As several kinds of antibody molecules have been developed by genetic engineering for disease treatment^17, 18^, it can solve the problem of cell specificity of siRNA. Compared to the IgG type antibodies, small antibodies, such as VHH, scFv, and antibody-like molecules, are expected to have stronger penetrating capability and lower immunogenicity^19^ due to the lack of both the constant region and Fc domain. Cell-penetrating peptides typically contain 5–30 amino acids and are mostly positively charged at physiological pH owing to the presence of several arginine and/or lysine residues^20^. These peptides can bind to nucleic acids, such as siRNA, through electrostatic interactions owing to their cationic nature. The arginine-9 (R9) motif, a typical cationic and cell-penetrating peptide, is more efficiently taken-up by cells than other oligomers containing fewer arginine residues or other cationic amino acids such as histidine, lysine, or ornithine^21^. By combining the technologies of cationic peptides and antibodies, successful cell-specific delivery of siRNA has been reported^15^. Moreover, no side effects were reported. However, although several cationic peptide-fused antibodies are functionally expressed as a soluble fraction^15, 16^, in many cases their functional expression was found to be very low in the bacterial host, and they were functionalized by unfolding the inclusion bodies^11–13^ or by bio-conjugating^14^. To expand this technology, it is necessary to develop a method for stable preparation of cationic peptide-fused antibodies.

An effective strategy for functional expression is to separate and express each functional domain and then fuse them in vitro^22^. This can effectively eliminate the complications related to structure and function as well as reduce undesirable non-expression and expression as an insoluble fraction. Reconstruction of each functional unit is generally carried out by bioconjugation using a polymer or particle as support; however, the production of well-defined antibody conjugates is extremely challenging owing to the required selectivity, specificity, and reactivity under physiological conditions^23^.

Recently, a peptide ligation method using sortase and transglutaminase has attracted attention as a method for specifically ligating proteins under mild conditions^24^. Recently, a VHH and CPP bioconjugate formed using sortase has been reportedly taken-up by cells^25, 26^. Microbial transglutaminase (MTG; EC 2.3.2.13), belonging to the class of a protein γ-glutamyl transferases, is the most widely used enzyme because of its high activity, Ca^+^ independence, and low reverse reaction^27^. The enzyme catalyzes the formation of an amide bond between the γ-carboxamide side chain group of glutamine residues as acyl donor and a primary ε-amine group of lysine as acyl acceptors. This enzyme can achieve specific ligation by adding peptides, such as K-tag sequence MRHKGS and Q-tag sequence LLQG, to the protein^28, 29^.

In this study, Nanobody-R9, which is not expressed as a fusion protein, was subjected to separate domain expression that were enzymatically ligated by MTG in vitro. Finally, the RNAi of a model siRNA created using the resulting Nanobody-R9^MTG^ was evaluated.

## Results

### Expression of Nanobody-R9

**Figure 1:**
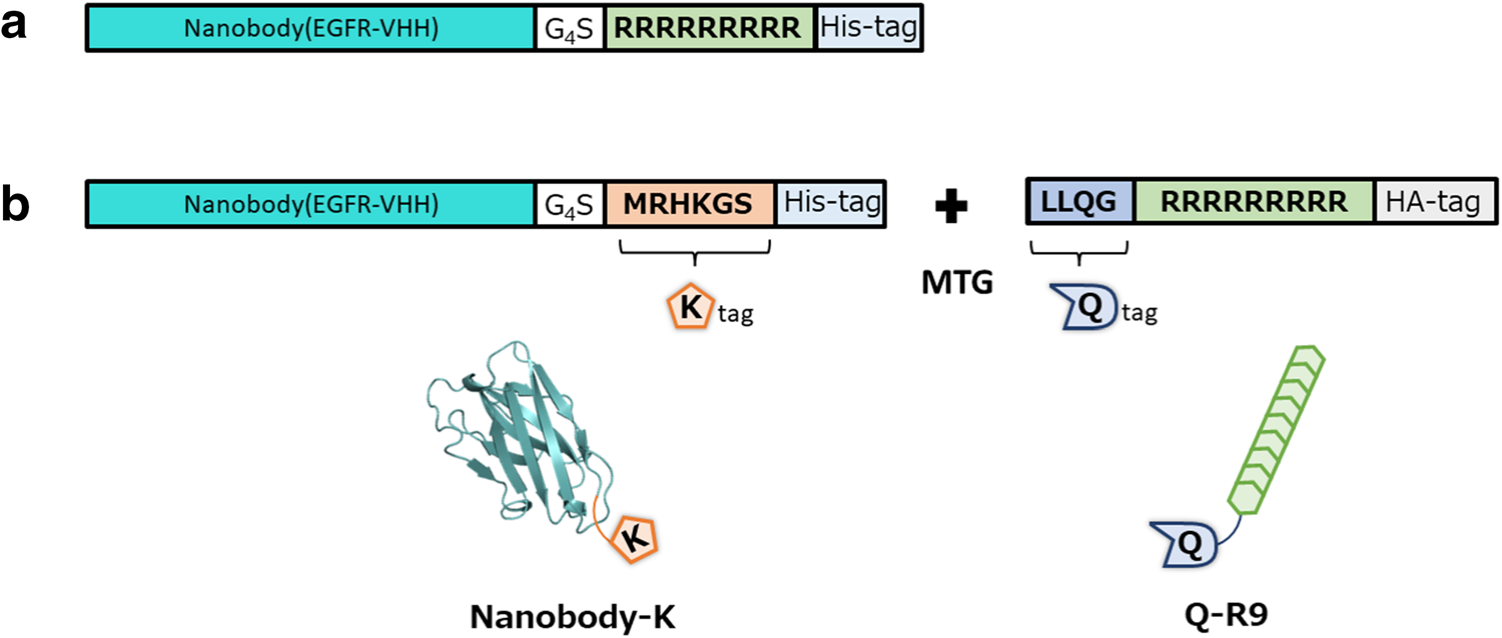
Primary structure of the protein designed in this study. (**a**) Nanobody-R9 (**b**) Nanobody-K and Q-R9-HA. Green color: Ia1 VHH (anti EGFR Nanobody), White color: G_4_S linker, Light green color: R9 peptide, Orange color: K-tag, Light blue color: His-tag, Purple color: Q-tag, and Glay color: HA-tag.

**Figure 2:**
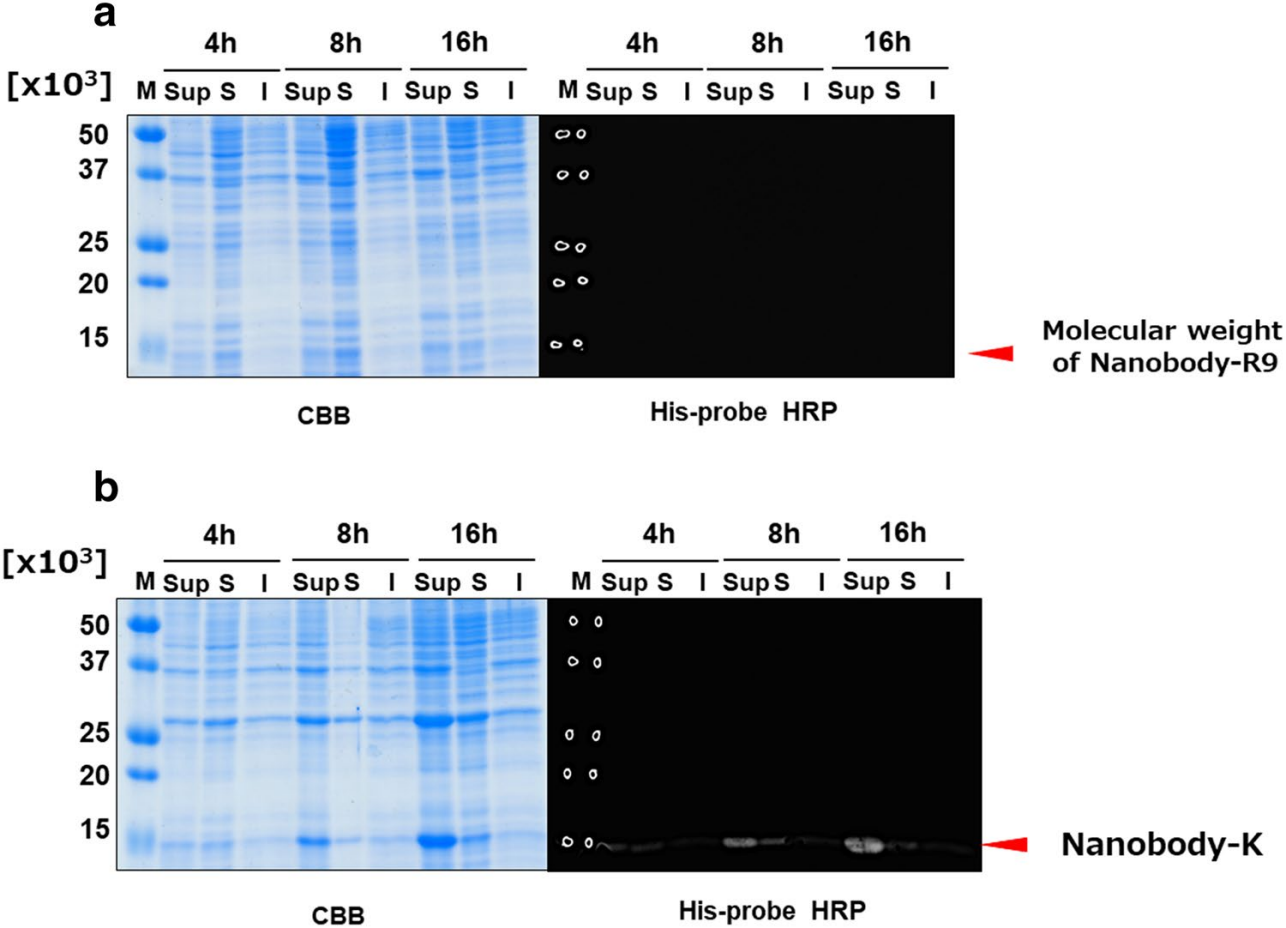
Expression analysis of Nanobody-R9 (**a**) and Nanobody-K (**b**) in *E. coli*. The figures show SDS-PAGE analysis (Left panels) using Coomassie Brilliant Blue (CBB) detection and western blot analysis (Right panels) using His-probe HRP detection of culture supernatant (Sup), intracellular soluble fraction (S), and intracellular insoluble fraction (I) under the induction at 28 °C for 4–16 h. M show Low molecular weight marker. The arrows indicate the deduced molecular weights of the target proteins. Complete gels and western blots are presented in Supplementary Fig. 1.

We selected an anti-epidermal growth factor receptor (EGFR) nanobody^30^, which is a low molecular weight VHH antibody fragment that can bind to the EGFR on cancer cell surface as a targeting molecule to enable cell-specific delivery of siRNA and the cationic peptide Arginine 9 (R9), a molecule that can interact with siRNA and penetrate the cell membrane. Using these, a gene coding anti-EGFR nanobody fused with R9 peptide at the C-terminus (Nanobody-R9) was constructed and genetically expressed in *E. coli* (Fig. 1a). Expression of the Nanobody-R9 gene was examined in the culture supernatant fraction, intracellular soluble fraction, and intracellular insoluble fraction post induction under conditions of different temperatures and isopropyl β-d-thiogalactopyranoside (IPTG) concentrations. Unfortunately, Nanobody-R9 was not expressed in any fraction (Fig. 2a, Supplementary Fig. S1).

### Enzymatic synthesis of Nanobody-R9^MTG^ using MTG

**Figure 3:**
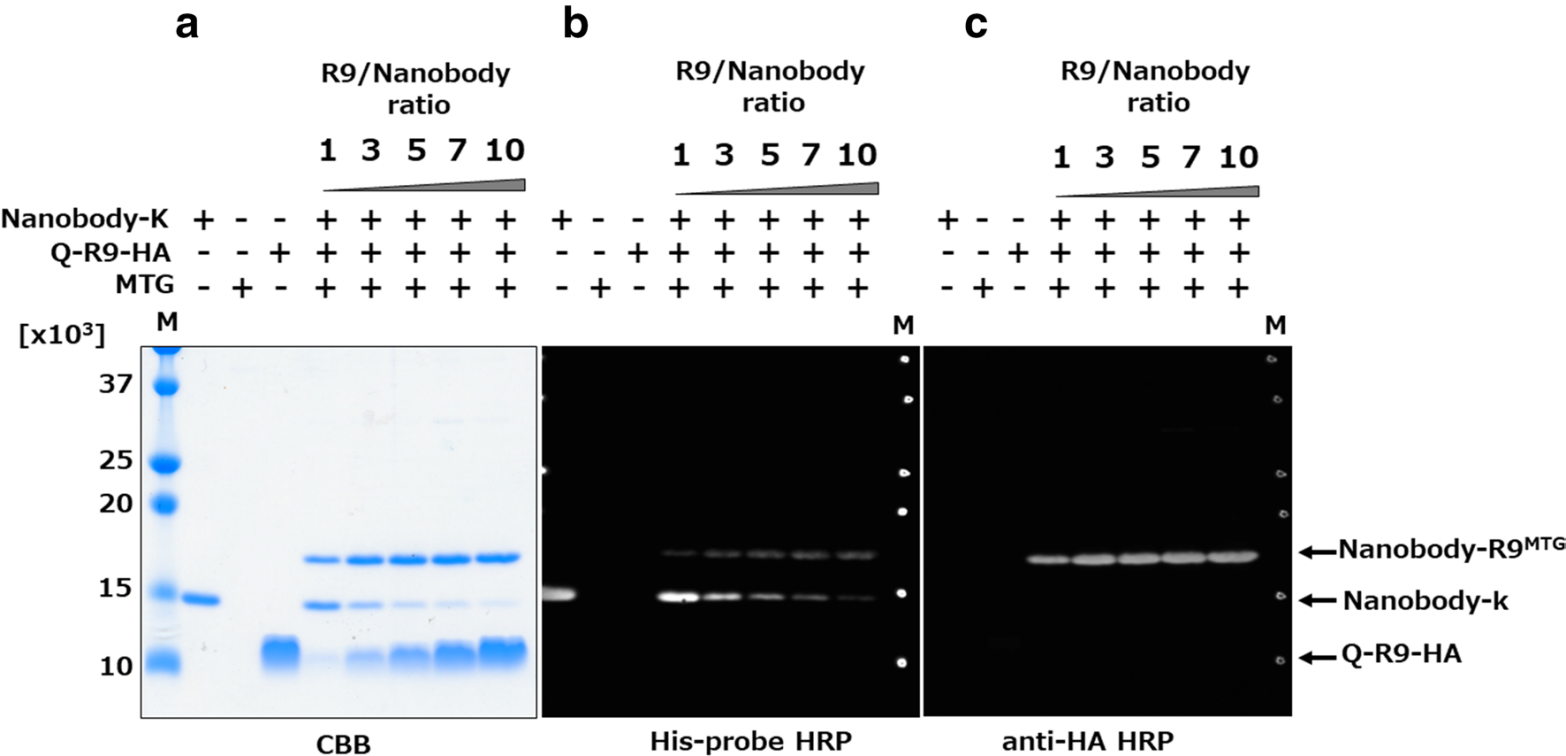
Enzymatic ligation of Nanobody and R9 by MTG. (**a**) SDS-PAGE by CBB staining; (**b**) western blot analysis using His-probe HRP; and (**c**) western blot analysis using anti-HA antibody. A total of 5–35 µM Q-R9-HA (1–10 times Nanobody concentration) was mixed with 5 µM Nanobody-K and 0.03 U mL^−1^ MTG and incubated at 20 °C for 6 h. The arrows indicate the deduced molecular weight of each protein. M indicate Low molecular weight marker for protein.

The Nanobody-R9 fusion protein was not expressed in *E. coli*. Therefore, we synthesized each nanobody and R9 separately and ligated them using MTG. Nanobody with K-tag, which is an MTG recognition sequence at the C-terminus (Nanobody-K, Fig. 1b) was expressed in *E. coli* as the intracellular soluble fraction (Fig. 2b). The Nanobody-K-expressing transformant was cultured for 16 h at 28 °C after IPTG induction, and Nanobody-K was purified using an immobilized metal-ion affinity chromatography (IMAC) and size-exclusion chromatography (SEC). The yield of purified Nanobody-K was calculated to be 27.0 mg L^−1^-broth. The purified Nanobody-K was ligated with an organically synthesized R9 fused Q-tag (Q-R9-HA), another MTG recognition tag, by MTG. Finally, 5 μM Nanobody-K was mixed with 1–10 times the molar amount (final 5–50 μM) of Q-R9-HA peptide using 50 nM of MTG purified by IMAC and SEC (15 U mg^−1^ protein). The mixture was incubated at 20 °C for 6 h. Individually, MTG, Q-R9-HA, and Nanobody-K did not show any product bands in SDS-PAGE analysis (Fig. 3a); however, their mixture showed a product band with a molecular weight of 18 × 10^3^. This molecular weight was similar to that of Nanobody-R9^MTG^ (19.1 × 10^3^), suggesting that Nanobody-R9^MTG^ was successfully prepared. Western blot analysis also showed that Nanobody-R9^MTG^ could be detected by HA-tag and His-tag (Fig. 3b,c), indicating that the product was a complex comprising Nanobody-K and Q-R9-HA. Notably, unreacted Q-R9-HA was not detected by HA-tag detection. Presumably, Q-R9-HA passes through the nitrocellulose membrane due to its small size during the transfer. The band size of Nanobody-R9^MTG^ increased slightly and that of unreacted Nanobody-K decreased in the reaction mixture with increasing concentration of Q-R9-HA. When the molar ratio of Nanobody:R9 was 1:1, 1:3, 1:5, 1:7, and 1:10, reaction efficiency of the nanobody was calculated to be 30.4, 62.7, 77.7, 83.0, and 85.1%, respectively, based on the signal intensity of band in SDS-PAGE.

To prove that MTG does not react with lysine residues in the nanobody sequence, we created a nanobody in which the K tag was replaced by a lysine-free Cmyc′ tag. No ligated product was found (Supplementary Fig. S2), indicating that MTG does not react with the nanobody and specifically reacts with the K-tag. The unreacted peptide and Nanobody-K in the solution after Nanobody-R9^MTG^ synthesis were removed by IMAC purification, and the purified Nanobody-R9^MTG^ was used in the subsequent experiments.

### Secondary structure analysis of Nanobody-R9^MTG^ using circular dichroism spectroscopy

**Figure 4:**
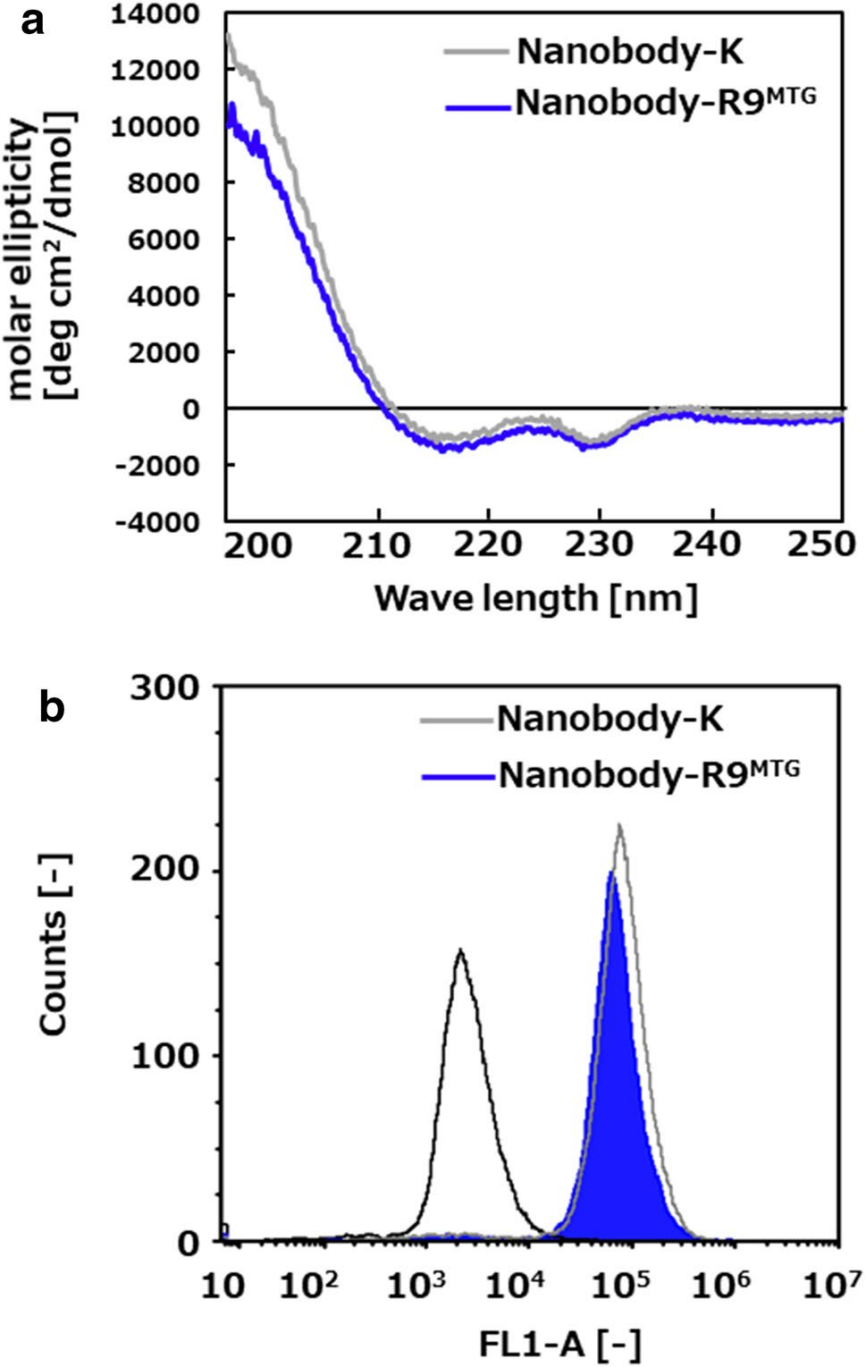
Physical properties of Nanobody-R9. (**a**) Analysis of Nanobody-R9^MTG^ and Nanobody-K secondary structure by CD spectra. (**b**) Analysis of binding affinity of Nanobody-R9^MTG^ and Nanobody-K by flow cytometry.

To confirm whether the Nanobody-R9^MTG^ retained its structure compared to the parent Nanobody-K, both structures were analyzed by circular dichroism spectrometry. As shown in Fig. 4a, the Nanobody-R9^MTG^ structure showed a typical β sheet-rich immunoglobulin fold, and no significant change was observed when compared with the structure of Nanobody-K. This result indicates that the nanobody part of Nanobody-R9^MTG^ formed a correct structure.

### Binding affinity of Nanobody-R9^MTG^ to EGFR

The binding affinity of Nanobody-R9^MTG^ to EGFR was evaluated to demonstrate that the crosslinking of R9 to nanobody did not affect the function of the nanobody. Nanobody-K and Nanobody-R9^MTG^, modified with FITC, were mixed with EGFR-positive A431 cells, and the fluorescent intensity of the interacting cells was analyzed by flow cytometry. Control A431 cells that were not treated with either nanobody showed a peak of fluorescence intensity of 2.5 × 10^3^ (Fig. 4b). Whereas, in case of nanobody-treated cells, both nanobodies were strongly bound to A431 cells. The peak fluorescence intensity for cells treated with Nanobody-R9^MTG^ (0.65 × 10^5^) was only slightly lower than that of cells treated with Nanobody-K (0.8 × 10^5^), indicating that there was no significant change in the binding affinity of the nanobody to EGFR after R9 crosslinking. We demonstrated that the nanobody module of Nanobody-R9^MTG^ is available to target EGFR-positive cells. The *K*d of Nanobody-R9^MTG^ was calculated as 63 ± 13 nM by flow cytometry analysis under various concentrations of the protein (Supplementary Fig. S3).

### Interaction of Nanobody-R9^MTG^ with siRNA

**Figure 5:**
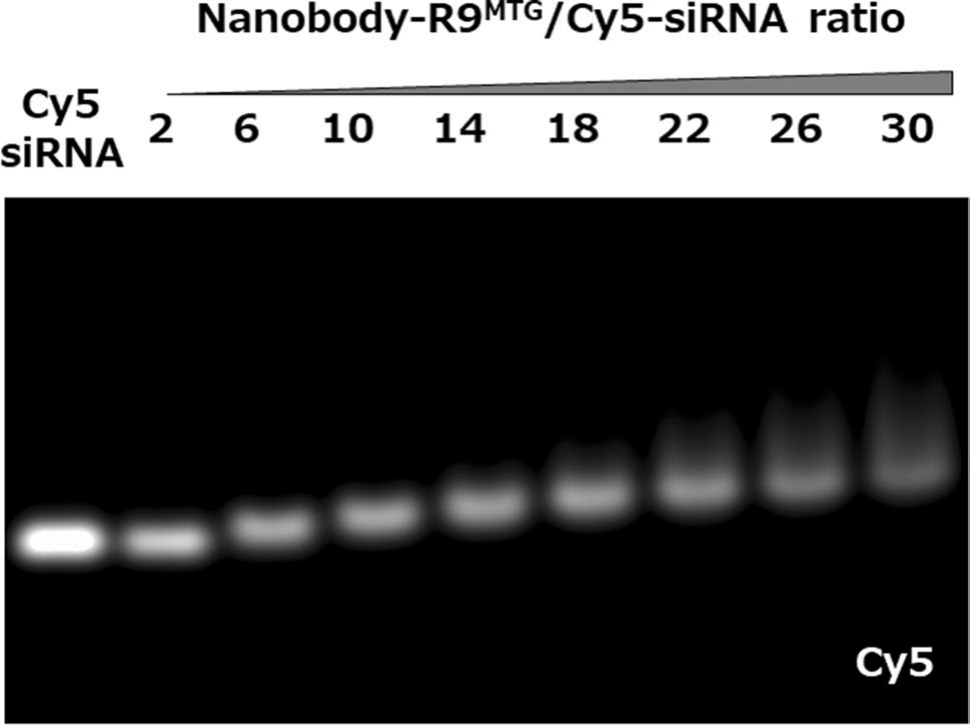
Interaction between siRNA and Nanobody-R9^MTG^. Electrophoretic mobility shift assay of Nanobody-R9^MTG^ and siRNA complex detected by Cy5. Full-length gel image provided in Supplementary Fig. S6.

The interaction between Nanobody-R9^MTG^ and siRNA was evaluated using an electrophoretic mobility shift assay. Briefly, 5 pmol Cy5-modified siRNA (Cy5-siRNA) and 10–150 pmol (2–30 times equivalent) Nanobody-R9^MTG^ were mixed at 20–25 °C for 30 min, and the reaction mixture was analyzed by agarose gel electrophoresis (Fig. 5). When Nanobody-R9^MTG^ concentration was at least sixfold higher than that of siRNA, the mobility of Cy5-siRNA decreased. This result showed that Cy5-siRNA interacted with Nanobody-R9^MTG^. Furthermore, the mobility of Cy5-siRNA decreased gradually with increase in ratio of Nanobody-R9^MTG^ to Cy5-siRNA, indicating that multiple Nanobody-R9^MTG^ molecules can bind to one siRNA. In addition, unreacted siRNA was not detected when using 6–30 times Nanobody-R9^MTG^ concentration, indicating that all siRNA interacted completely with Nanobody-R9^MTG^. Thus, we demonstrated the availability of the R9 peptide in Nanobody-R9^MTG^ to capture siRNA.

### siRNA delivery into EGFR-positive cells with Nanobody-R9^MTG^

**Figure 6:**
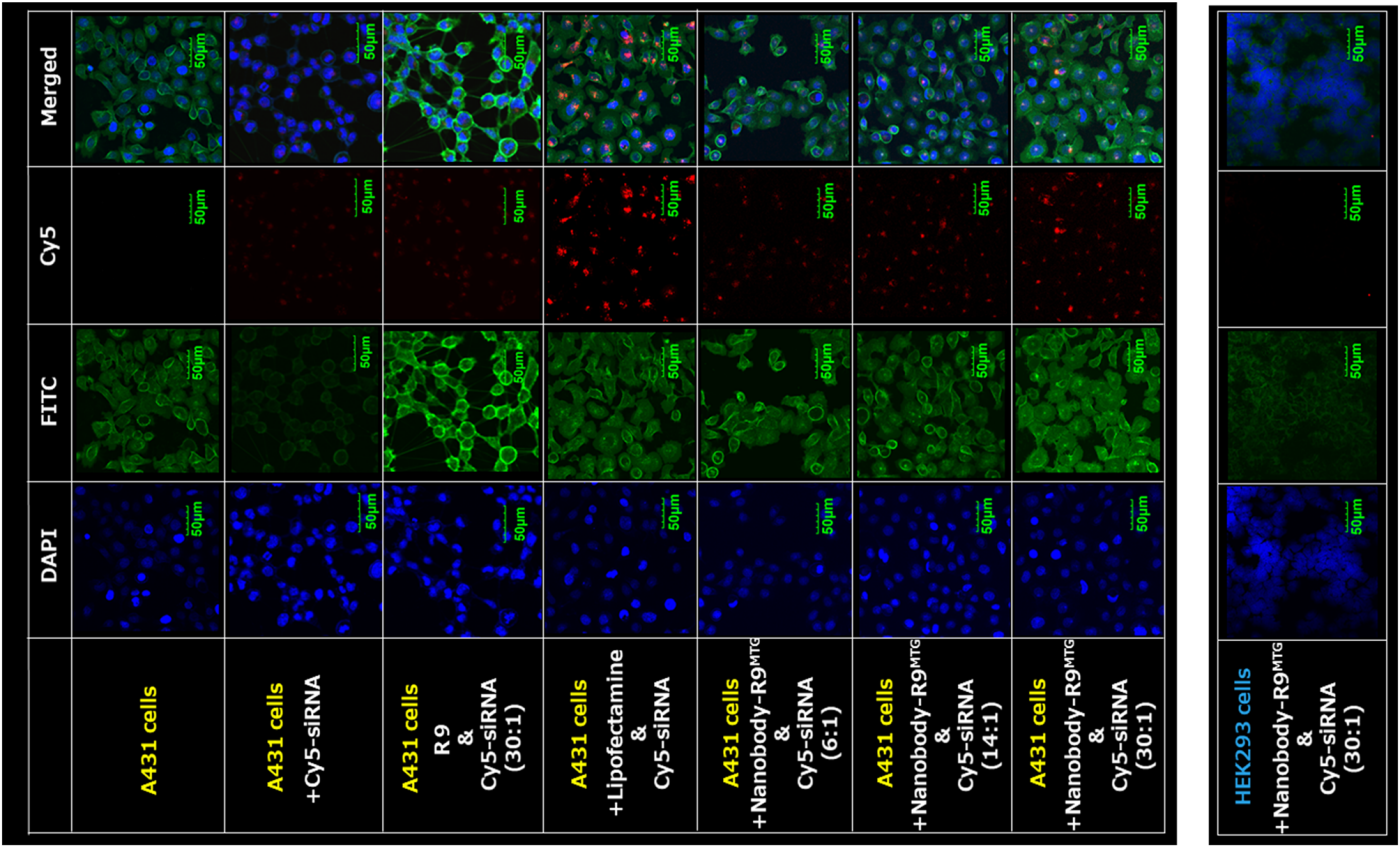
Delivery of siRNA by Nanobody-R9^MTG^ into A431 cells. EGFR positive cells (A431) were mixed with Nanobody-R9^MTG^-Cy5 siRNA. Cell nuclei and membranes were stained with DAPI and Cell Mask™ Green Plasma Membrane stain. The fluorescence of the cells after the reaction was observed using a confocal microscope. HEK293 cells were used as EGFR negative cell. Lipofectamine is used as positive control.

We demonstrated, using confocal microscopy, that siRNA could be successfully delivered into EGFR-positive cells using Nanobody-R9^MTG^ (Fig. 6). After incubating the final 20 nM Cy5-siRNA and its 6-, 14-, and 30-fold Nanobody-R9^MTG^ concentration for 30 min at 20–25 °C, these mixtures were added to the pre-cultured A431 cells and incubated at 37 °C in 5% CO_2_. After incubation for 6 h, cell nuclei and cell membranes were pre-stained with DAPI, and Cell Mask Green Plasma in the cell was observed by confocal microscopy. We used Lipofectamine 2000, a high-efficiency DNA and RNA transfection reagent, as a positive control. We found that when siRNA alone was added to the cells, trace amount of Cy5 fluorescence was observed in the cells; whereas, when Nanobody-R9^MTG^ and siRNA were added to the cells, siRNA was detected strongly in the cells under all conditions. This result showed that the addition of Nanobody-R9^MTG^ improved the efficiency of siRNA delivery. In addition, the fluorescence intensity of Cy5 increased with increase in the concentration of Nanobody-R9^MTG^, indicating that the siRNA is more likely to be taken up into cells under conditions of Nanobody-R9^MTG^ multivalency. Moreover, Nanobody-R9^MTG^ and siRNA complex at 30:1 ratio entered in the cell more than R9 and siRNA complex at 30:1 ratio, indicating that conjugation of nanobody to R9 is effective for introduction into specific cells. Under all conditions, the uptake efficiency of the Nanobody-R9^MTG^–siRNA complex by cells was lower than that of the positive control Lipofectamine. However, the addition of Nanobody-R9^MTG^–siRNA to EGFR-negative HEK293 cells under the optimal conditions did not deliver siRNA into the cells (Supplementary Fig. S4).

### RNAi with Nanobody-R9^MTG^–siRNA complex

**Figure 7:**
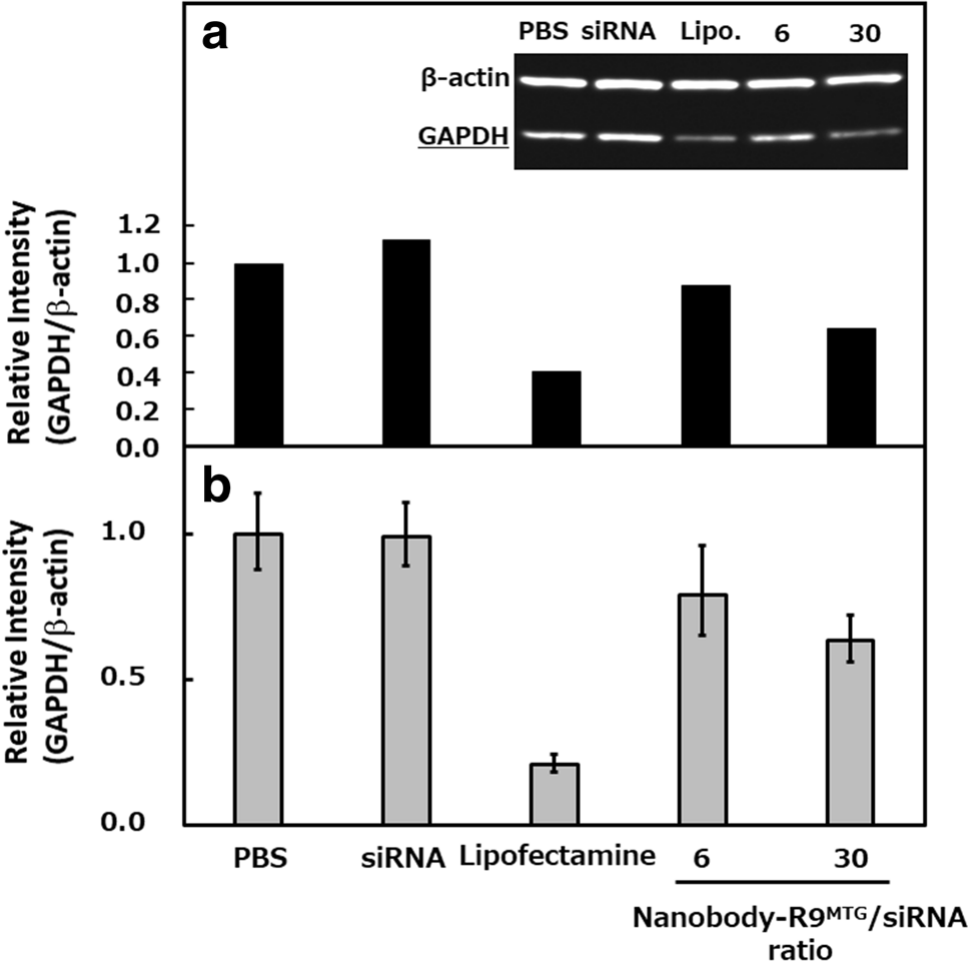
RNAi by Nanobody-R9^MTG^–siRNA. (**a**) Western blot analysis of GAPDH in the A431 cell extract mixed with Nanobody-R9^MTG^**–**siRNA using anti-GAPDH monoclonal antibody. (**b**) Real time PCR of the transcript in A431 cell extract mixed with Nanobody-R9^MTG^**–**siRNA. Lipofectamine was used as a positive control. *PBS* phosphate buffered saline, *Lipo* lipofectamine, 6 and 30 indicate the Nanobody-R9^MTG^/siRNA ratio. T-test showed less than 0.05 for all combinations except between PBS and siRNA (T-test < 0.54). Full-length gel image for (**a**) provided in Supplementary Fig. S7.

To investigate whether the Nanobody-R9^MTG^–siRNA complex can suppress the production of the target protein, a Nanobody-R9^MTG^–siRNA complex targeting glyceraldehyde-3-phosphate dehydrogenase (GAPDH) as a model was prepared and RNAi was evaluated (Fig. 7). Twenty picomoles of siRNA was mixed with 120 or 600 pmol of Nanobody-R9^MTG^ and the complex was added to cultured A431 cells. After 72 h, the amount of GAPDH in the cell extract was detected by western blot analysis with an anti-GAPDH antibody (Fig. 7a). When siRNA alone was used, the GAPDH production was not changed compared to that of β-actin, a housekeeping protein. However, when the Nanobody-R9^MTG^–siRNA complex was used, the production of GAPDH was significantly reduced. This result indicates that the RNAi of the specific transcripts was significantly more efficient when using the Nanobody-R9^MTG^–siRNA complex than that with siRNA alone. GAPDH production was calculated based on the band intensity in western blot as a percentage of GAPDH expression compared to β-actin expression. As a result, the production levels of GAPDH in 6- and 30-fold samples were 87.8% and 64.6%, respectively, indicating that they were suppressed by 12.2% and 35.4%, respectively. This performance was not as good as that of Lipofectamine (60.4%). Moreover, we confirmed the decreased target protein production due to silencing of its RNA using RT-PCR (Fig. 7b). We found that the amount of the transcripts correlated with the signal intensity of western blot analysis. Thus, GAPDH production was affected by RNAi-mediated transcriptional repression.

## Discussion

### Construction of fusion protein using transglutaminase

In recent years, researchers have actively focused on cell-specific oligonucleotide therapeutics using antibodies that have fewer side effects. However, one of the obstacles to developing an antibody–siRNA complex as cell-specific oligonucleotide therapeutic is the difficulty in functional expression of a fusion protein comprising an antibody and cell-penetrating peptide in *E. coli*. Therefore, it is necessary to establish a stable antibody–cell-penetrating peptide fusion method. Here, we solved this problem by enzymatic fusion using MTG. We focused on the nanobody (VHH)-fused R9 peptide that is not expressed in *E. coli* but is expected to induce tissue-specific RNAi as a fusion protein. Nanobodies are recombinant single variable domain antibodies derived from heavy-chain only antibodies that have feature such as small size (less than 15 kDa), excellent solubility, great stability, short blood half-life, high affinity and specificity for target^31^. Nanobodies closely resemble human VH sequences, and Ackaert et al. demonstrated that two non-humanized Nbs are candidates with a low immunogenicity risk profile^32^. In addition, Nanobodies can be reduced more in immunogenicity by being humanized. In recent years, Caplacizumab, a Nanobody format-based drug, has been launched for the first time as nanobody, proving that it can break through the approval barrier. In the future, this modality will be advanced in various projects^33^. In the present study, the antibody and R9 were prepared separately and expressed successfully in *E. coli*. Although the underlying mechanism is not clear, the fusion of R9 peptide to antibody appears to restrict its expression in *E. coli*. In the present study, the K-tag peptide did not prevent expression, at least in the R9 peptide. Similar examples of easy expression due to functional domain division have been previously reported in a wide range of proteins, including enzymes and antibodies^34, 35^. The domain separating process for *E. coli* expression was applied to generate Nanobody-R9. MTG efficiently catalyzed the enzymatic ligation of the antibody and R9. The reaction was completed by simply mixing the two at 20 °C with PBS as the solvent.

Nanobody-R9^MTG^ production increased with increase in molar ratio of Q-R9-HA to Nanobody-K and plateaued at when the ratio of Nanobody-K:Q-R9-HA was 1:7. The reaction efficiency, based on band intensity, under optimum conditions was calculated to be 85.1% when the ratio of Nanobody-K:Q-R9-HA was 1:10. On the other hand, with increasing dosage of R9 peptide, the amount of unreacted R9 increased. As a result, at the molar ratio of 1:10, 90% of R9 remained unreacted. However, Nanobody-R9 and residual R9 peptides could be easily removed by gel filtration chromatography. Moreover, the R9 peptide was recovered from the reaction mixture and could be reused (Supplementary Fig. S5). In addition, the structure of Nanobody did not change before and after peptide ligation; the fused product showed almost the same function as that of the unfused nanobody. In this design, the domains were linked via a G_4_S linker to avoid steric hindrance. Our design was successful and worked well in this study.

### Binding of Nanobody-R9 to siRNA and delivery of siRNA to cells

Nanobody-R9 interacted with siRNA and the results showed that not only Nanobody, but also R9, was functional after enzymatic ligation by MTG. Several Nanobody-R9 molecules could bind to a single siRNA molecule. Even though the valence of Nanobody-R9 exceeded 30, it continued to increase. Such a tendency has also been reported in previous studies^14, 20, 36^. The cells were incubated Nanobody-R9–siRNA containing two different molar ratios (1:6, 1:30) of siRNA to Nanobody-R9. siRNA alone did not enter the cell, showing its low cell-penetration efficiency because the negative charges of the cell membrane and the siRNA repel each other^37^. However, the Nanobody-R9–siRNA can facilitate the entry of the siRNA into the EGFR-positive cells, albeit in smaller amounts than that facilitated by R9-siRNA under the same concentration conditions. This result showed that the entry of R9 into cells was accelerated by the Nanobody. We believe that this improvement in uptake efficiency is due to EGFR-dependent endocytosis, as stated in previous reports^20^. Moreover, we found that with increasing valency of Nanobody-R9 to siRNA, the amount of Nanobody-R9–siRNA entering the cells increased. Finally, the amount of Nanobody-R9 transferred into the cells was approximately 50% that of Lipofectamine 2000 under optimal conditions. This result is consistent with previous reports^20^. This increased uptake frequency was attributed to the increased affinity of nanobodies to EGFR due to the multivalent effect, which facilitates EGFR-dependent endocytosis. Along similar lines, a previous study reported that the rate and level of antibody internalization largely depends on the affinity of the engineered antibodies towards FGFR1, as high-affinity antibodies display the fastest internalization kinetics^38^. Furthermore, we suspect that not only EGFR endocytosis but also R9-induced macropinocytosis may have an effect on uptake frequency. Wang et al. reported that R9 peptide and siRNA bound, through electrostatic interaction, at a charge ratio of about 1:12 (1:56 molar ratio), resulting inefficient delivery of siRNA into cells^36^. The net charge of Nanobody R9 would be biased towards the positive charge beyond cancellation of the negative charge of siRNA under the optimal conditions for entering a cell. Electrostatic interaction with the membrane of Nanobody-R9–siRNA induces macropinocytosis^39^.

### RNAi with Nanobody-R9^MTG^

Using the Nanobody-R9 and GAPDH siRNA, we investigated RNAi induction; GAPDH production was reduced by adding Nanobody-R9^MTG^–siRNA, proving that RNAi could be triggered. We constructed a molecule that facilitated GAPDH siRNA transfer into the nucleus, specifically silencing the GAPDH transcript, and finally suppressed 35.4% GAPDH protein production. Lu et al. have previously reported that the addition of scFv-R9/Her2 siRNA suppressed ~ 45% of Her2 expression, whereas scFv/Her2 siRNA did not alter Her2 expression^40^. Examination at the transcriptional level revealed similar results, suggesting that the reduction in transcript level was related to a reduction in protein production. Moreover, increasing the valency of R9 on siRNA increased the effect of RNAi. siRNA has to interact with RNA induced silencing complex (RISC) to work in the nucleus or cytoplasm. Increasing the valency of R9 may enhance the proton sponge effect proposed by Boussif et al.^41^; they reported that the amino group of the cationic peptide that had been deprotonated extracellularly (pH 7.4) is first protonated inside the late endosome (pH ~ 5.5). Thereafter, siRNA escape would occur due to counterion influx and osmotic pressure increase, resulting in endosomal destabilization. We hypothesize that endosomal escape with proton sponge effect is triggered by increase in amino group of arginine with internalization of a large amount of R9 in the present study.

In conclusion, the fusion protein Nanobody-R9, which is not expressed under normal conditions, was expressed separately as individual functional domain and then ligated with MTG to successfully express the fusion protein. The coupling efficiency reached 85.1%. Our study provides a basis for the generation of such functional fusion proteins that have many potential applications but are not expressed in an expression system.

## Methods

### Materials

siRNAs: Silencer Select GAPDH Positive Control siRNA was purchased from Thermo Fisher Scientific (San Jose, CA, USA). These siRNAs were used after labeling with Cy5 using the *Label* IT siRNA Tracker Cy5 Kit (Mirus Bio LLC, Madison, WI, USA). Peptides: The synthetic peptide Q-tag-R9-HA (5′-Acetyl-LLQGRRRRRRRRRYPYDVPDYA-COOH-3′) was purchased from Eurofins Genomics (Ebersberg, Germany). Cell lines: A431 human epidermoid carcinoma cells were obtained from the Cell Resource Center for Biomedical Research, Institute of Development, Aging, and Cancer, Tohoku University (Sendai, Japan). HEK293 cells were obtained from the Japanese Collection of Research Bioresources Cell bank (Osaka, Japan). These cells were cultured in RPMI 1640 medium with L-glutamine and sodium bicarbonate (liquid, sterile-filtered, suitable for cell culture; Sigma Aldrich, St. Louis, MO, USA) supplemented with 10% fetal bovine serum (Biowest, France) and 1 × Anti-Anti (Gibco, Thermo Fisher Scientific, Waltham, MA, USA).

### Construction of expression vector

The gene encoding anti-EGFR nanobody from Ia1 llama antibody was amplified to fuse the K-tag-His-tag (GGGGSMRHKGSHHHHHH), R9-His-tag, (GGGGSRRRRRRRRRHHHHHH), or Cmyc′-His-tag (TMFLISEEDLQHHHHHH) to its C-terminus by polymerase chain reaction (PCR). PCR products and pRA expression vector were digested with NcoI and SpeI in case of K-tag-His-tag and R9-His-tag, or with NcoI and SacII in case of Cmyc′-tag, and ligated to each other at 16 °C for 30 min to generate pRA-Nb-K-His, pRA-Nb-R9-His, and pRA-Nb-Cmyc′-His expression vectors.

The gene encoding MTG from *Streptomyces mobaraensis* (accession number DQ132977) fused 6 × His-tag to the C-terminus synthesized by Thermo Fisher Scientific and was amplified by PCR to generate NcoI and EcoRI sites. PCR products and the pET22b expression vector were digested with NcoI and EcoRI, followed by ligation at 16 °C for 30 min to generate the pET22b (+)-MTG-His expression vector.

### Expression and purification of recombinant antibodies

*Escherichia coli *BL21 (DE3) transformants harboring pRA-Nb-K-His, pRA-Nb-R9-His, and pRA-Nb-Cmyc′-His were cultured at 28 °C in flasks containing 2 × YT medium supplemented with 100 µg mL^−1^ ampicillin, and protein expression was induced by adding 1 mM IPTG when the absorbance of the culture at 600 nm reached 0.8. After incubation at 28 °C for 16 h, the culture supernatant and intracellular soluble and insoluble fractions were collected and evaluated by SDS-PAGE and western blot. Proteins were purified using IMAC (Ni Sepharose 6 Fast Flow; GE Healthcare Bio-Sciences AB, Uppsala, Sweden) and SEC (HiLoad 26/600 Superdex 75 prep grade; GE Healthcare Bio-Sciences AB).

### Expression and purification of MTG

Transformed *E. coli* BL21 (DE3) cells harboring expression plasmids encoding MTG were incubated in 2 × YT medium containing 100 g mL^−1^ ampicillin at 37 °C, and expression of recombinant antibodies under the control of the T7 promoter was induced by adding 0.4 mM IPTG when the absorbance of the culture at 600 nm was 0.8. After additional incubation at 20 °C for 30 h, the bacterial supernatant was collected and purified using IMAC and SEC (HiLoad 26/600 Superdex 200 prep grade; GE Healthcare Bio-Sciences AB).

### MTG-mediated fusion of Nanobody-K and Q-R9 and purification of Nanobody-R9^MTG^

The fusion of each nanobody and R9 peptide was conducted by mixing 5 µM Nanobody-K (or Nanobody-Cmyc′), 5–35 µM Q-R9, and 0.03 U mL^−1^ MTG in PBS (pH 7.4) at 20 °C for 6 h. The reaction product was analyzed by SDS-PAGE and western blot analysis, and reaction efficiency was evaluated based on band intensity using Image Quant Las 4000 (GE Healthcare Bio-Sciences AB). The reaction product was purified using IMAC (1 mL His trap HP; GE Healthcare Bio-Sciences AB) with 0–400 mM imidazole gradient in 1 × PBS containing 750 mM NaCl to remove MTG and unreacted Q-R9 and Nanobody-K.

### Electrophoretic mobility shift assay

Nanobody-R9^MTG^ and 5 pmol Cy5-siRNA were mixed, with increasing amount of Nanobody-R9 in PBS. The mixture was incubated at 4 °C for 30 min and then electrophoresed on 2% (w/v) agarose gels in TAE buffer. The mobility shift of the siRNA band was visualized on Cy5 detected using a UV-transilluminator.

### Flow cytometry

The specific binding of Nanobody-R9^MTG^ or Nanobody-K to EGFR on the cell surface was analyzed by flow cytometry. For this, forty-seven nM of Nanobody-R9^MTG^ or Nanobody-K were labeled with Fluorescein Labeling kit-NH_2_ (Dojindo, Inc., Kumamoto, Japan) and mixed with 2 × 10^6^ EGFR-positive A431 cells. The mixtures were incubated for 60 min on ice. The cells were washed three times with PBS containing 0.01% bovine serum albumin and analyzed by flow cytometry (FACS Accuri 6; BD Biosciences, Franklin Lakes, NJ, USA).

### Confocal microscopy

A431 cells (1.5 × 10^5^) were grown on Glass bottom dishes (Matsunami Glass Ind., Ltd., Japan) in RPMI medium for 24 h at 37 °C and 5% CO_2_. Then, 50 pmol Cy5-siRNA was mixed with 300 or 1500 pmol Nanobody-R9^MTG^ in PBS (final volume 500 µL), and the mixture was incubated at 4 °C for 60 min. Lipofectamine 2000 (Thermo Fisher Scientific, Japan) was used as a positive control. The reaction mixture was added to the culture medium of A431 cells followed by incubation at 37 °C for 6 h in the cell incubator. The cells were washed twice with PBS and stained with 1 mg mL^−1^ 4′,6-diamidino-2-phenylindole (DAPI; Sigma-Aldrich, Japan) and Cell Mask Green plasma Membrane stain (FITC detection, Thermo Fisher Scientific, Japan). After staining, the cells were observed under a confocal microscope (FV1200-D; Olympus, Japan).

### Western blot

A431 cells (1.5 × 10^4^) were grown on 24-well plates in RPMI medium for 24 h at 37 °C. Then, 20 pmol Cy5-siRNA was mixed with 120 or 6000 pmol Nanobody-R9^MTG^ in PBS (final volume 150 µL) and incubated at 4 °C for 60 min. Lipofectamine 2000 was used as a positive control. The mixture was added to the culture medium of A431 cells and incubated at 37 °C for 72 h in a cell incubator. Thereafter, the cells were washed in PBS and lysed directly with RIPA buffer (Nacalai Tesque, Kyoto, Japan). The extracted proteins were then transferred onto nitrocellulose membranes (Millipore, Bedford, MA, USA) that were incubated with primary antibodies against GAPDH (1:7500; MBL, Nagoya, Japan) and β-actin (1:7500; MBL), followed by incubation with horseradish peroxidase (HRP)-conjugated goat anti-mouse Ig secondary antibody (1:7500; Proteintech Group, Inc., Tokyo, Japan). Finally, Chemi-Lumi One (Nacalai Tesque) was used to visualize the protein bands.

### Real time PCR

First, 120 or 6000 pmol Nanobody-R9^MTG^ was mixed with 20 pmol siRNA in 150 µL of PBS and incubated at 4 °C for 60 min. A431 cells (3.0 × 10^4^) were grown on 24-well plates (Costar 24 well 3524, Corning, NY, USA) in RPMI medium at 37 °C overnight. The cells were then washed in PBS and incubated with 250 mL Nanobody-R9 siRNA (with a final concentration of 40 nM siRNA) in 250 µL RPMI medium at 37 °C for 24 h. Thereafter, cells were washed three times in PBS. Next, the cells were lysed using SingleShotCell Lysis kit (Bio-Rad laboratories, Inc., CA, USA) and cDNA was synthesized using Advanced cDNA synthesis kit for RT-PCR (Bio-Rad laboratories, Inc.) according to the manufacturer’s instructions. RT-PCR of GAPDH transcripts was performed using a mixture of 1 µL cDNA, 5 µL 5 × iScript SYBR Green supermix (Bio-Rad laboratories, Inc.), 10 pmol µL^−1^ GAPDH forward primer: 5′-GTCTCCTCTGACTTCAACAGCG-3′ and GAPDH reverse primer: 5′-ACCACCCTGTTGCTGTAGCCAA-3′ and nuclease-free water by thermal cycling under following conditions: 40 cycles of 95 °C for 30 s, 95 °C for 10 s, and 60 °C for 30 s. The cDNA content was calculated using the ΔΔCt method with Actin as a housekeeping gene (Actin forward primer: 5′-CACCATTGGCAATGAGCGGTTC-3′ and Actin reverse primer: 5′-AGGTCTTTGCGGATGTCCACGT-3′.

## Supplementary Information

Supplementary Information.

## Abbreviations

IMAC: Immobilized metal ion affinity chromatography
scFv: Single-chain variable fragments
SEC: Size exclusion chromatography
EGFR: Epidermal growth factor receptor
SDS-PAGE: Sodium dodecyl sulfate polyacrylamide gel electrophoresis
MTG: Microbial transglutaminase
