# Supplementary material for: Enzymatic ligation of an antibody and arginine 9 peptide for efficient and cell-specific siRNA delivery
Source: Sci Rep. 2021 Nov 8;11:21882. doi: 10.1038/s41598-021-01331-1 (PMC8575896; doi:10.1038/s41598-021-01331-1)
Supplement: Supplementary file 1 — Supplementary Information. [file 41598_2021_1331_MOESM1_ESM.docx]

**Supporting Information**

**Enzymatic ligation of an antibody and arginine 9 peptide for efficient and cell-specific siRNA delivery**

Yu Ando, Hikaru Nakazawa^*^, Daisuke Miura, Maho Otake, Mitsuo Umetsu^*^

**Affiliation:** Department of Biomolecular Engineering, Graduate School of Engineering, Tohoku University, Sendai, Japan.

*Corresponding author:

**Mitsuo Umetsu**

E-mail: mitsuo@tohoku.ac.jp

**Hikaru Nakazawa**

E-mail: hikaru@tohoku.ac.jp

Phone number +81-22-795-7276

**Supplementary Information**


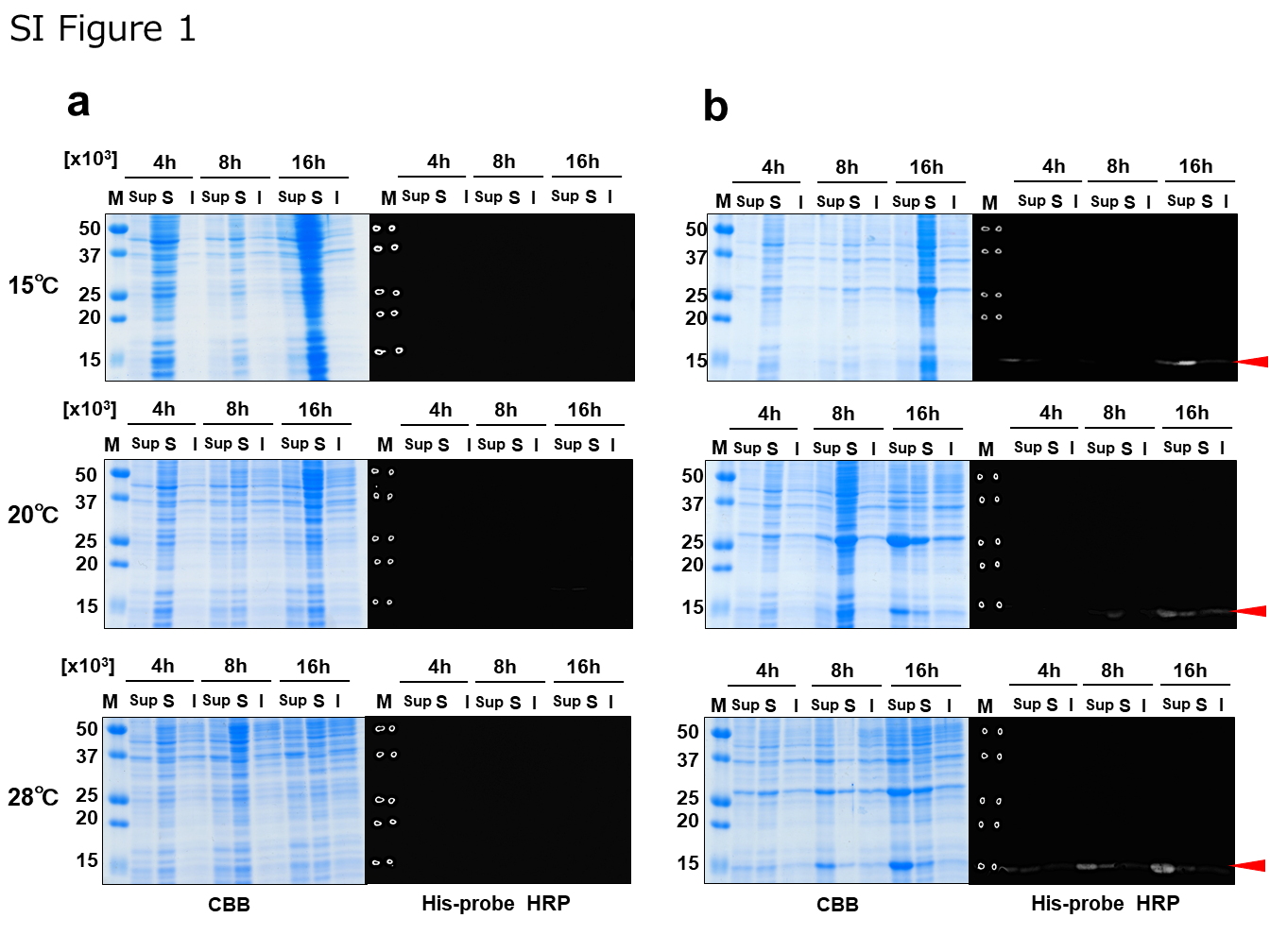


**Figure S1. (a) Expression analysis of Nanobody-R9 and (b)Nanobody-K in *E. coli*.**

*E. coli* BL21 (DE3) transformants harboring pRA-Nb-K-His, pRA-Nb-R9-His were cultured at 28 °C in flasks containing 2×YT medium supplemented with 100 μg mL^-1^ ampicillin, and protein expression was induced by adding 1 mM IPTG when the absorbance of the culture at 600 nm reached 0.8. After incubation at 15°C, 20°C, and 28 °C for 4-16 h, the culture supernatant, intracellular soluble fractions and intracellular insoluble fractions were collected and evaluated by SDS-PAGE and western blot.

Each left panel shows SDS-PAGE analysis by CBB staining. Right panel shows western blot analysis using His-probe HRP. M: Low molecular weight marker, Sup: culture supernatant, S: intracellular soluble fraction, I: intracellular insoluble fraction. The arrows indicate the deduced molecular weights of the target proteins.


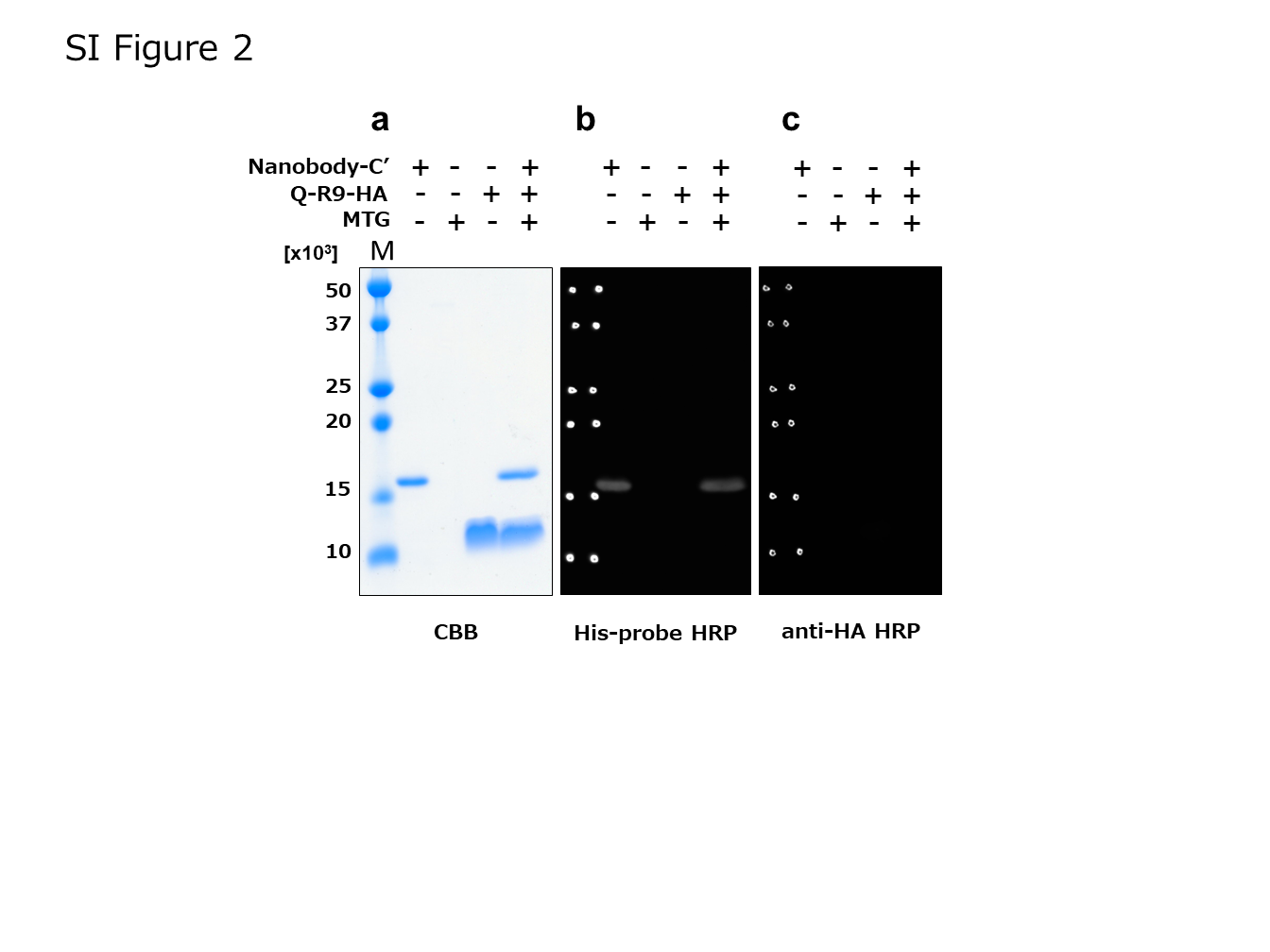


**Figure S2. Enzymatic ligation of Nanobody-Cmyc' and R9 by MTG**

(a) SDS-PAGE by CBB staining; (b) western blot analysis using His-probe HRP; and (c) western blot analysis using anti-HA antibody. A total of 5–35 µM Q-R9-HA (1–10 times Nanobody concentration) was mixed with 5 mM Nanobody-C' (Nanobody**-**Cmyc') and 0.03 U mL^-1^ MTG and incubated at 20°C for 6 h. M indicate Low molecular weight marker for protein. Because Cmyc has no lysine residue VHH and R9 did not connect.


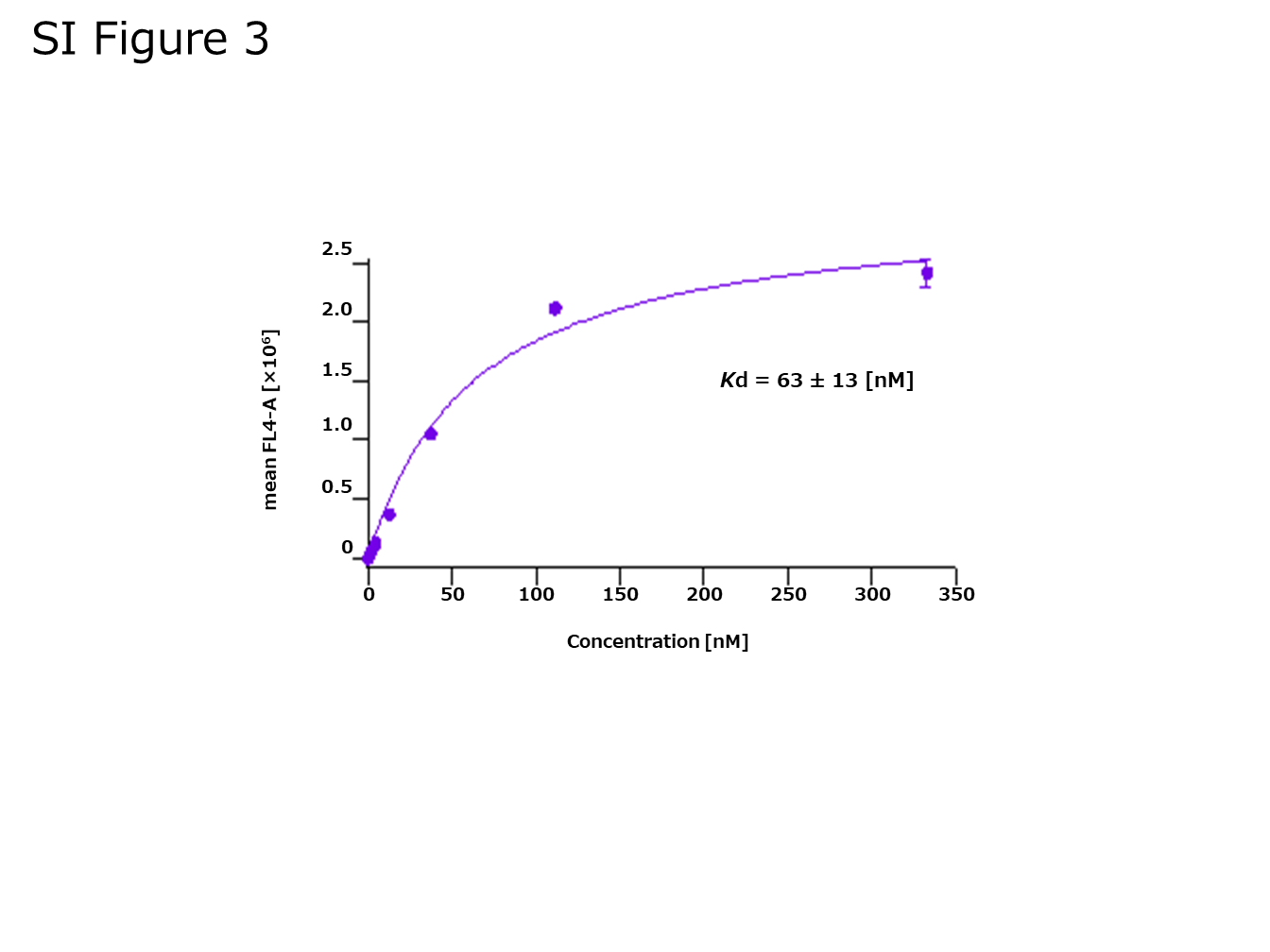
**Figure S3. Calculation of *K*d value of Nanobody-R9^MTG^ by flow cytometry.**


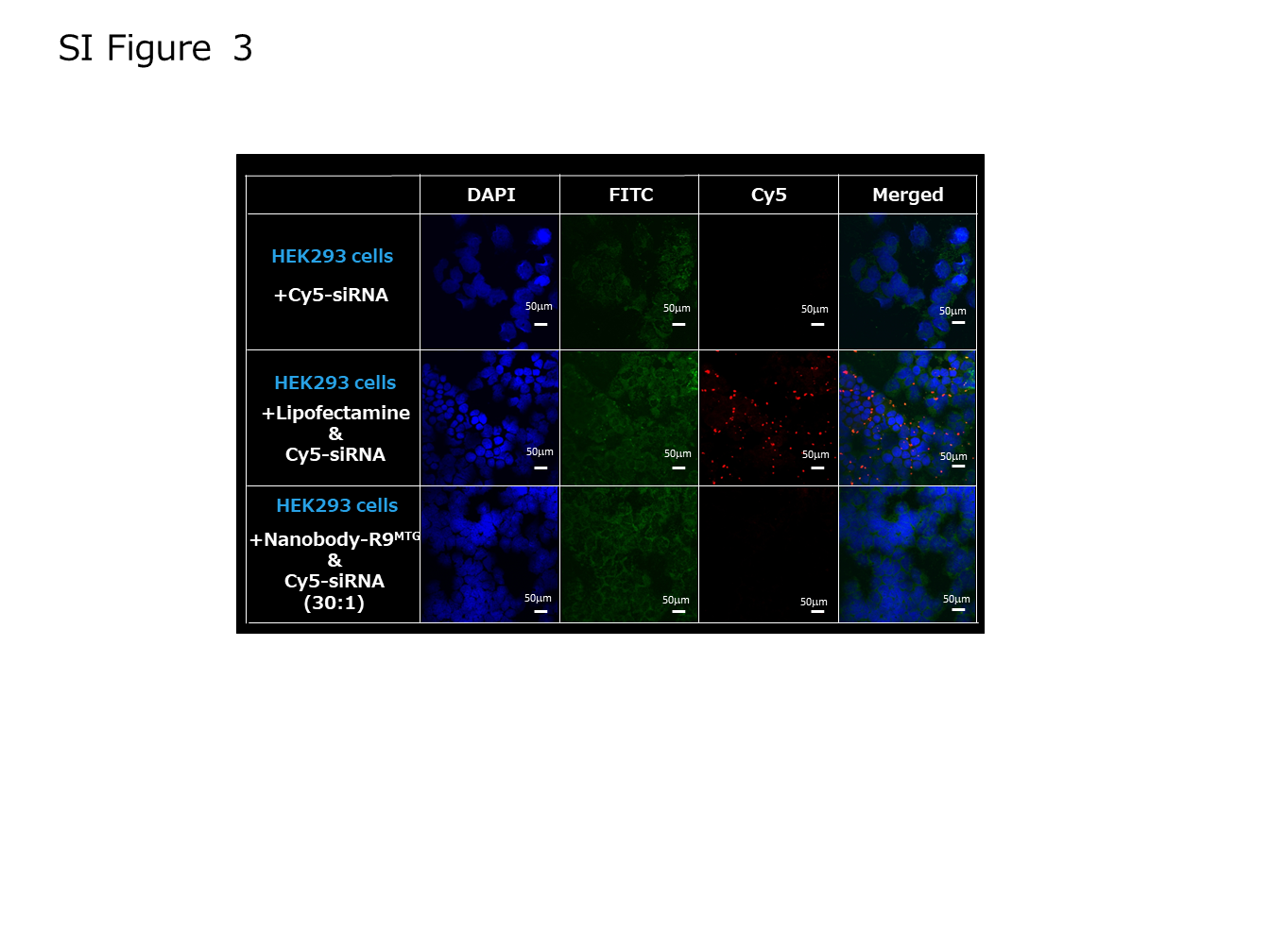


**Figure S4.** **Delivery of siRNA by Nanobody-R9^MTG^ into HEK293 cells.**

EGFR negative cells (HEK293) were mixed with Nanobody-R9^MTG^-Cy5 siRNA. Cell nuclei and membranes were stained with DAPI and Cell Mask™ Green Plasma Membrane stain. The fluorescence of the cells after the reaction was observed using a confocal microscope. Lipofectamine is used as positive control.


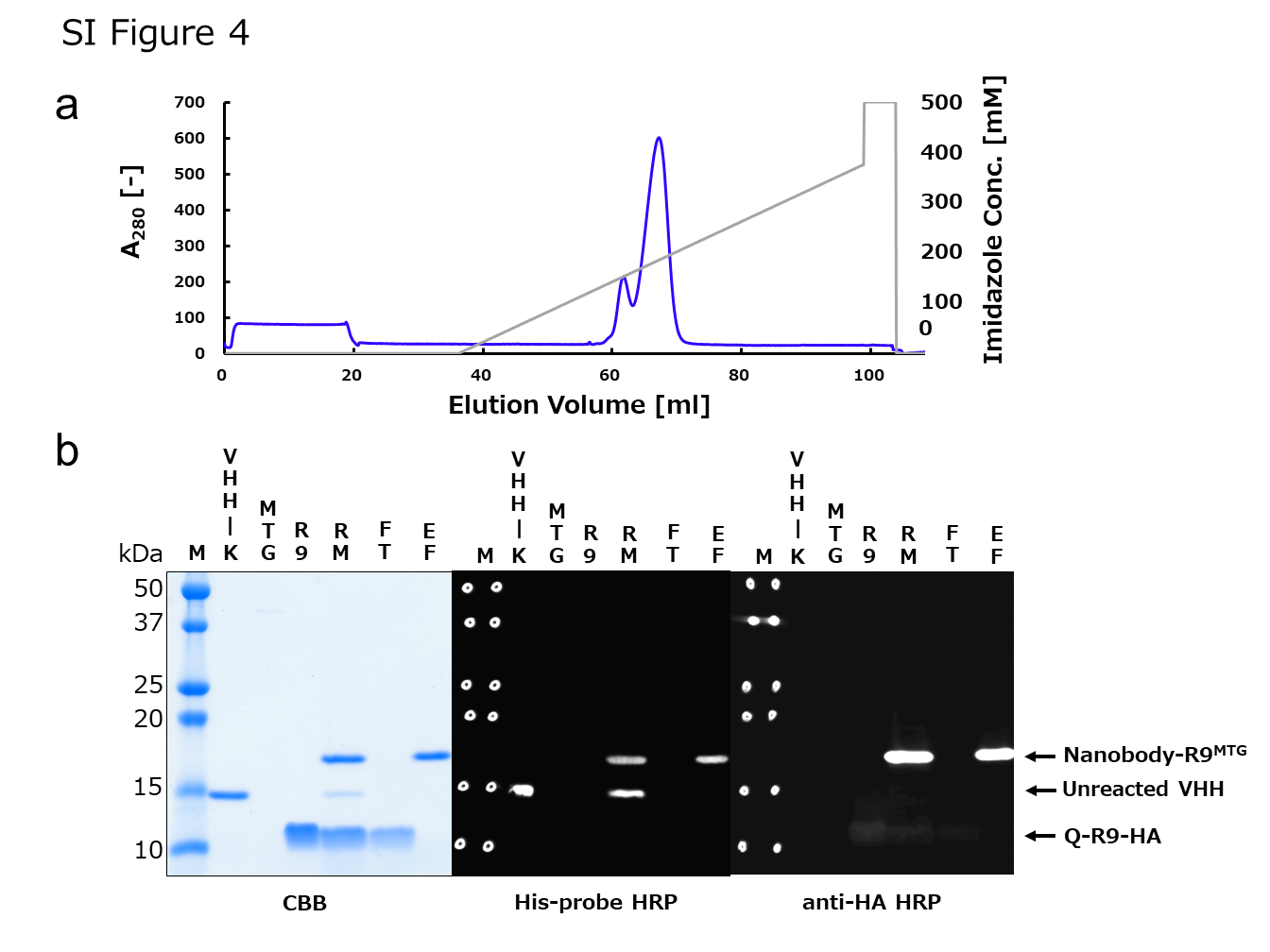


**Figure S5. Separation of Nanobody-R9^MTG^ and R9 peptide by IMAC.**

(a) Chromatography chart for reaction mixture by Histrap HP with 0-400mM Imidazole gradient. (b) SDS-PAGE and western analysis of separation of Nanobody-R9^MTG^ and R9 peptide. RM: Reaction mixture, FT: Flowthrough Fraction, E: Elution Fraction.


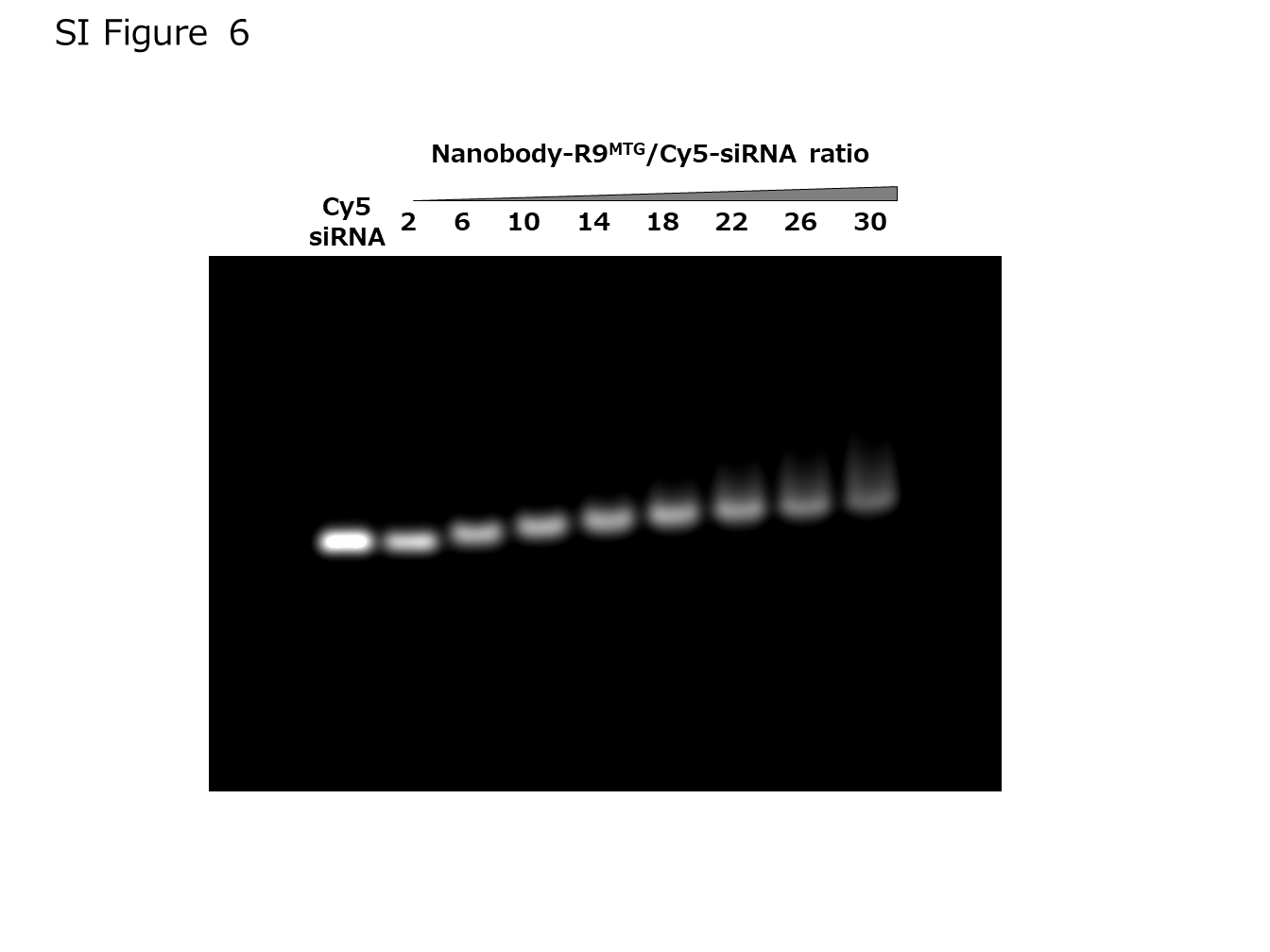


**Cy5**

**Figure S6. Interaction between siRNA and Nanobody-R9^MTG^.**


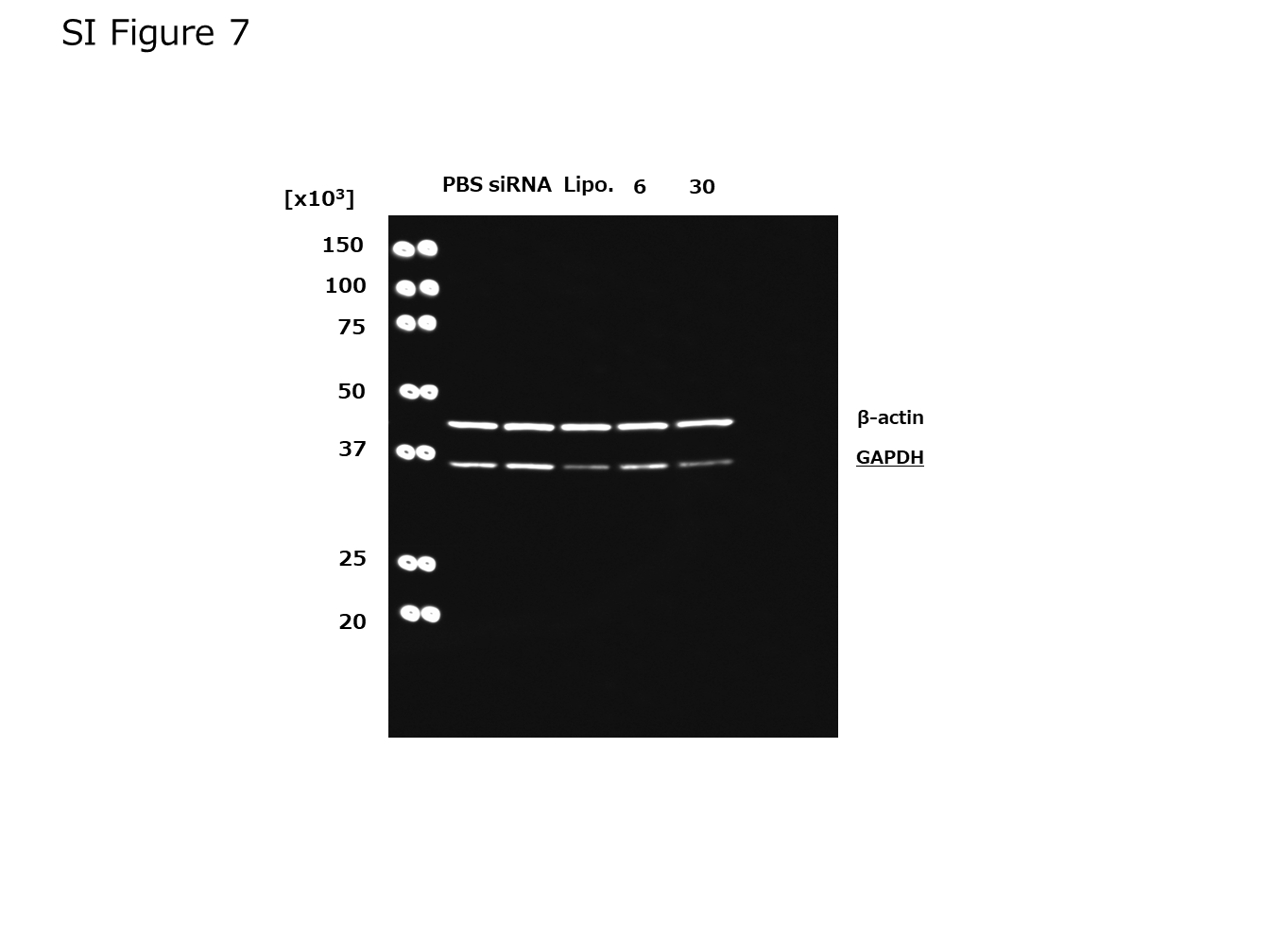


**Figure S7. Western blot analysis of GAPDH in the A431 cell extract mixed with Nanobody-R9^MTG^–siRNA using anti-GAPDH monoclonal antibody.**
